# Preparation and Property Characterization of In_2_YSbO_7_/BiSnSbO_6_ Heterojunction Photocatalyst toward Photocatalytic Degradation of Indigo Carmine within Dye Wastewater under Visible-Light Irradiation

**DOI:** 10.3390/ma15196648

**Published:** 2022-09-25

**Authors:** Jingfei Luan, Bowen Niu, Bingbing Ma, Guangmin Yang, Wenlu Liu

**Affiliations:** 1 School of Physics, Changchun Normal University, Changchun 130032, China; 2 State Key Laboratory of Pollution Control and Resource Reuse, School of the Environment, Nanjing University, Nanjing 210093, China

**Keywords:** In_2_YSbO_7_, In_2_YSbO_7_/BiSnSbO_6_ heterojunction photocatalyst, indigo carmine, photocatalytic degradation, visible-light irradiation, N-doped TiO_2_

## Abstract

In_2_YSbO_7_ and In_2_YSbO_7_/BiSnSbO_6_ heterojunction photocatalyst were prepared by a solvothermal method for the first time. The structural characteristics of In_2_YSbO_7_ had been represented. The outcomes showed that In_2_YSbO_7_ crystallized well and possessed pyrochlore constitution, a stable cubic crystal system and space group *Fd3m*. The lattice parameter of In_2_YSbO_7_ was discovered to be a = 11.102698 Å and the band gap energy of In_2_YSbO_7_ was discovered to be 2.68 eV, separately. After visible-light irradiation of 120 minutes (VLGI-120M), the removal rate (ROR) of indigo carmine (IC) reached 99.42% with In_2_YSbO_7_/BiSnSbO_6_ heterojunction (IBH) as a photocatalyst. The ROR of total organic carbon (TOC) reached 93.10% with IBH as a photocatalyst after VLGI-120M. Additionally, the dynamics constant k which was taken from the dynamic curve toward (DCT) IC density and VLGI time with IBH as a catalyst reached 0.02950 min^−1^. The dynamics constant k which came from the DCT TOC density and VLGI time with IBH as a photocatalyst reached 0.01783 min^−1^. The photocatalytic degradation of IC in dye wastewater (DW) with IBH as a photocatalyst under VLGI was in accordance with the first-order kinetic curves. IBH was used to degrade IC in DW for three cycles of experiments under VLGI, and the ROR of IC reached 98.74%, 96.89% and 94.88%, respectively, after VLGI-120M, indicating that IBH had high stability. Compared with superoxide anions or holes, hydroxyl radicals possessed the largest oxidative ability for removing IC in DW, as demonstrated by experiments with the addition of trapping agents. Lastly, the probable degradation mechanism and degradation pathway of IC were revealed in detail. The results showed that a visible-light-responsive heterojunction photocatalyst which possessed high catalytic activity and a photocatalytic reaction system which could effectively remove IC in DW were obtained. This work provided a fresh scientific research idea for improving the performance of a single catalyst.

## 1. Introduction

In the last few years, the water pollution problem had drawn worldwide attention. Among the number of pollutants, organic pollutants derived from dye wastewater (DW)from textile and photography industries became an acute environmental problem due to its unacceptable color, high-chemical oxygen demand, toxicity and biodegradation. Indigo carmine (IC) could be detected in dyes, and IC was widely used in food, medicine, clothing and other fields. Moreover, IC was carcinogenic in nature and could cause serious health problems in human beings, including reproduction, neurons, acute toxicity, eye or skin exposure, hypertension, cardiovascular or respiratory problems [1]. The organic pollutants from DW could be removed by coagulation–flocculation, adsorption and membrane filtration. However, because of the disadvantages of toxicity and high price [2,3], many macromolecular organic pollutants could not be biodegraded. The photocatalysis technique which possesses the strengths of environmental conservation, low cost and high treatment efficiency [4,5] is widely used in treating sewerage. From the perspective of energy, photocatalytic technology is more scientific and promising because photocatalytic technology only requires the use of sunlight for activating catalysts. [6]. Photocatalysts can effectively decompose organic pollutants because of the production of the oxidation free radicals. This is a new interdisciplinary research field. Catalysts can be regenerated and recycled, which is of great significance for the research work of photocatalysis technology in the future. 

By analyzing previous reports, it was found that a large number of metal oxides such as TiO_2_ and ZnO [7,8] have been developed as photocatalysts. However, the application of a single photocatalyst was limited because of intrinsic properties such as photo-etching and wide band gaps [9,10]. TiO_2_ could only absorb ultraviolet rays effectively, but ultraviolet light energy only occupies 5% account of solar energy; as a result, light energy was not fully utilized. There was a great breakthrough when Zou found that Ni-doped InTaO_4_ compounds were responsive to wavelengths in the visible-light region in 2001 [11]. Zou demonstrated that the development of visible-light-responsive photocatalysts for optimal use of visible-light energy, which occupied 43% of sunlight energy, was possible. Researchers were inspired to explore new photocatalysts, which is reflected in the extensive efforts of former scholars toward achieving degradation of pollutants under visible-light irradiation (VLGI). Therefore, the key to photocatalytic technology was the development of the photocatalysts that possess high catalytic efficiency [12,13,14,15,16]. There are many methods which have been proven to be effective, for example, ion doping methods such as N-doped TiO_2_, the heterojunction construction method [17,18,19,20,21,22,23] and the photosensitization method [24,25]. The higher light utilization efficiency of the composite material system [22,23,24,25,26,27,28] reflected that the composite material system contained various functions of a single photocatalyst [29,30,31,32,33], higher photocatalytic performance, longer carrier life and higher chemical stability [34,35,36,37,38,39,40].

It is common knowledge within the field that slight changes in the internal structure of the photocatalyst can affect the photocatalytic activity. Luckily, A_2_B_2_O_7_ compounds are known for their photocatalytic performance under VLGI. Xing et al. [41,42] prepared Bi_2_Sn_2_O_7_ compound and Y_2_Ti_2_O_7_ compound with a A_2_B_2_O_7_ structure, which possessed good photocatalytic performance under VLGI. Based on our previous work [43], we found that Sm_2_FeSbO_7_ had a pyrochlore structure. As a catalyst under VLGI, the structural metamorphosis of Sm_2_FeSbO_7_ could hold potential for improving photocatalytic activity. According to the above analysis, we suppose that stead of Sm^3+^ by In^3+^, and stead of Fe^3+^ by Y^3+^ in Sm_2_FeSbO_7_ might increase carrier concentration. According to the above analysis, we may conclude that the structure and properties of the new In_2_YSbO_7_ compound can be changed and improved, and more advanced photocatalytic properties can be obtained.

During the process of photocatalysis, heterojunction catalysts have excellent performance [44,45,46]. The heterojunction could greatly improve the electron transfer rate and redox performance of the catalyst by inhibiting electron–hole recombination within two semiconductors [47]. Sabzehparvar et al. [48,49,50,51] prepared a battery of heterojunction catalysts with excellent properties, such as TiO_2_/NiO-Ag heterojunction catalyst, MnTiO_3_/TiO_2_ heterojunction catalyst, AgBr/BiPO_4_ heterojunction catalyst and Bi_2_MoO_6_/Bi_4_V_2_O_11_ heterojunction catalyst. Subsequently, these catalysts showed better performance during the degradation of organic pollutants in DW. The heterojunction catalysts which were prepared by Channei et al. all showed good effects on the degradation of indigo in DW; the heterojunction catalysts used were WO_3_/CeO_2_ catalyst, MoS_2_/Cu_2_O catalyst and ZnBi_2_O_4_/ZnS catalyst [52,53,54]. Analysis indicated that the synthesis of heterojunction photocatalysts could improve the redox performance of photocatalysts [55] and improved the total selectivity and reactivity. In conclusion, the heterojunction structure of the catalysts is a promising research direction.

The structural properties of pure-phase BiSnSbO_6_ and In_2_YSbO_7_ prepared by a solvothermal method were analyzed. The transmission electron microscopy (TEM) was utilized for analyzing the structural properties of nitrogen-doped TiO_2_ (N-TiO_2_). Moreover, the removal rate (ROR) of indigo carmine (IC) under VLGI with In_2_YSbO_7_ as a catalyst or with BiSnSbO_6_ as a catalyst or with N-TiO_2_ as a catalyst or with In_2_YSbO_7_/BiSnSbO_6_ heterojunction (IBH) as a catalyst was discovered. The novel research content of this article was to prepare a novel In_2_YSbO_7_ nanocatalyst and In_2_YSbO_7_/BiSnSbO_6_ heterojunction photocatalyst (IBHP) for the first time. A photocatalyst that responded to visible light with high photocatalytic activity was acquired, and could degrade IC efficiently. Degradation of organic pollutants in DW with IBH as a catalyst showed higher efficiency and stronger security.

## 2. Results and Discussion

### 2.1. XRD Analysis

**Figure 1:**
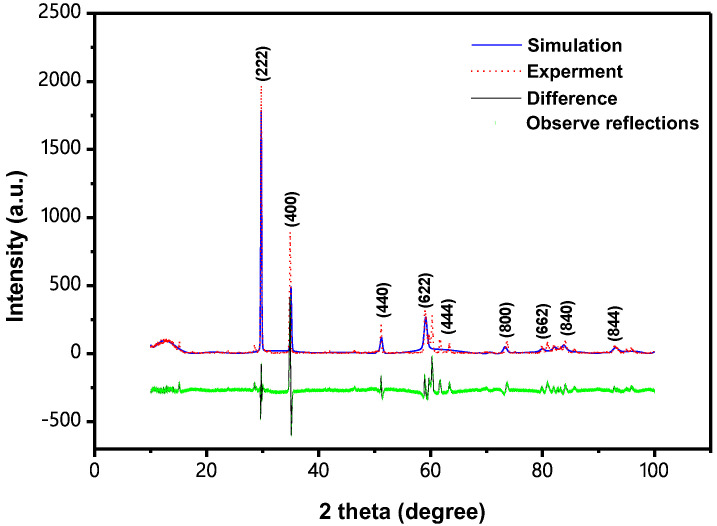
XRD pattern and Rietveld refinement of In_2_YSbO_7_ (red dotted line represents experimental XRD data for In_2_YSbO_7_; blue solid line represents simulated XRD data for In_2_YSbO_7_; black solid line represents the difference between experimental and simulated XRD data for In_2_YSbO_7_; green vertical lines indicate the observed reflection locations).

**Figure 2:**
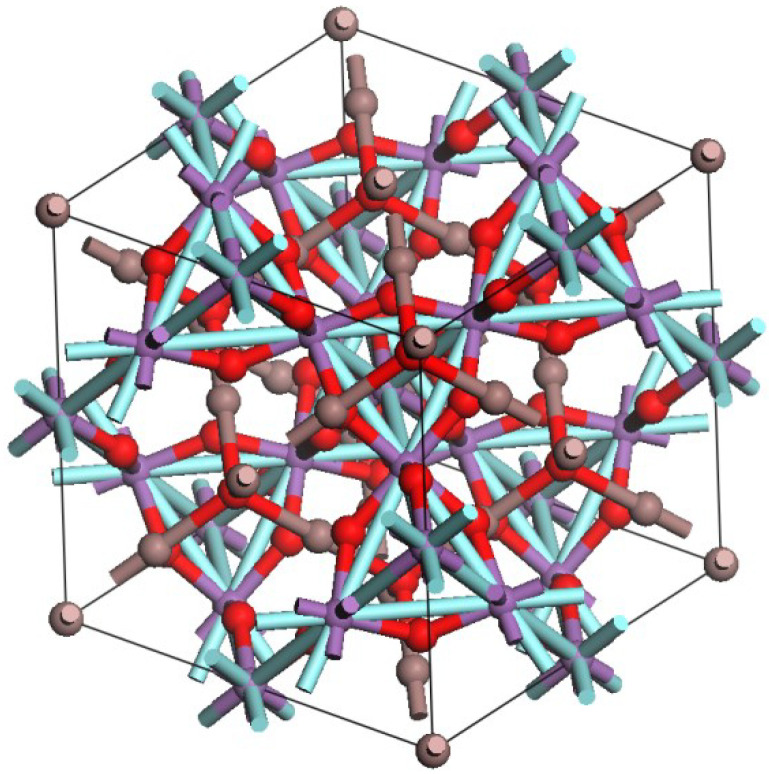
Atomic structure of In_2_YSbO_7_. (Red atom: O; cyan atom: In; purple atom: Y or Sb.).

Structure of the prepared In_2_YSbO_7_ uses X-ray diffraction technology for detection and the results are shown in Figure 1. By analyzing the results, it was shown that In_2_YSbO_7_ was a single phase and the lattice parameter of the new catalyst In_2_YSbO_7_ was 11.102698 Å. Additionally, the whole of the diffraction peaks for In_2_YSbO_7_ could be indexed smoothly on the principle of the lattice constant and the above space group *Fd3m*. Table 1 shows the atomic coordinates and structural parameters of In_2_YSbO_7_. Figure 2 shows the atomic structure of In_2_YSbO_7_. It could be concluded from Figure 1 that In_2_YSbO_7_ crystallized into a pyrochlore-type structure. The structure of In_2_YSbO_7_ was refined, and the results showed that the unweighted R-factor R_P_ was 44.31% and the obtained space group was *Fd3m*. 

The x-coordinate of the known O (1) atom could be used as an indicator of the crystal structure change of pyrochlore-type A_2_B_2_O_7_ compound (cubic system, space group *Fd3m*) and if six A–O (1) bonds were the same length as two A–O (2) bonds, both equal to 0.375 [56]. The value of x for MO_6_ (M = Y^3+^ and Sb^5+^) deviated from x = 0.375 [56]; thus, the distortion of the MO_6_ (M = Y^3+^ and Sb^5+^) octahedron was evident in the crystal structure of In_2_YSbO_7_. Charge separation was required for photocatalytic degradation (PCD) of IC under VLGI to prevent the recombination of photoinduced electrons (PE) and photoinduced holes (PH). Inoue [57] and Kudo [58] showed that the local distortion of the MO_6_ octahedron, which came from catalysts such as BaTi_4_O_9_ and Sr_2_M_2_0_7_ (M = Nb^5+^ and Ta^5+^), was necessary in preventing recombination between charges and contributed to the amelioration of photocatalytic activity. Similarly, in the crystal structure of In_2_YSbO_7_, the (M = Y^3+^ and Sb^5+^) octahedral distortion of MO_6_ was also considered to contribute to the enhanced photocatalytic activity. In_2_YSbO_7_ consisted of a three-dimensional network of octahedra with a corner sharing MO_6_ (M = Y^3+^ and Sb^5+^). The MO_6_ (M = Y^3+^ and Sb^5+^) octahedra were linked into chains by In^3+^ ion. Two kinds of In–O bond lengths (BL) coexist: six In–O (1) BL (2.636Å) were significantly longer than 2 In–O (2) BL (2.230Å). The six M–O (1) (M = Y^3+^ and Sb^5+^) BL were 1.939 Å and the M–In (M = Y^3+^ and Sb^5+^) BL were 3.642 Å. The M–O–M (M = Y^3+^ and Sb^5+^) bond angle (BA) was 139.624° in the crystal structure of In_2_YSbO_7_. The In–M–In (M = Y^3+^ and Sb^5+^) BA was 135.000° in the crystal structure of In_2_YSbO_7_. The In–M–O (M = Y^3+^ and Sb^5+^) BA was 135.505° in the crystal structure of In_2_YSbO_7_. The study of luminescent properties showed that the angle between MO_6_ (M = Y^3+^ and Sb^5+^) octahedra, such as the M–O–M BA of In_2_YSbO_7_, had an important influence on the photocatalytic activity of In_2_YSbO_7_. The closer the M–O–M BA was to 180°, the greater the mobility of PE and PH was; as a result, the photocatalytic activity was stronger because the mobility of PE and PH affected the probability of electrons and holes reaching the reaction sites on the catalyst surface [59].

Furthermore, the Sb–O–Sb BA of In_2_YSbO_7_ was larger, which led to an increase in photocatalytic activity of In_2_YSbO_7_. By analyzing the above results, under VLGI conditions with In_2_YSbO_7_ as a catalyst, the effect of IC degradation was mainly attributed to the crystal structure and electronic structure of In_2_YSbO_7_.

**Figure 3:**
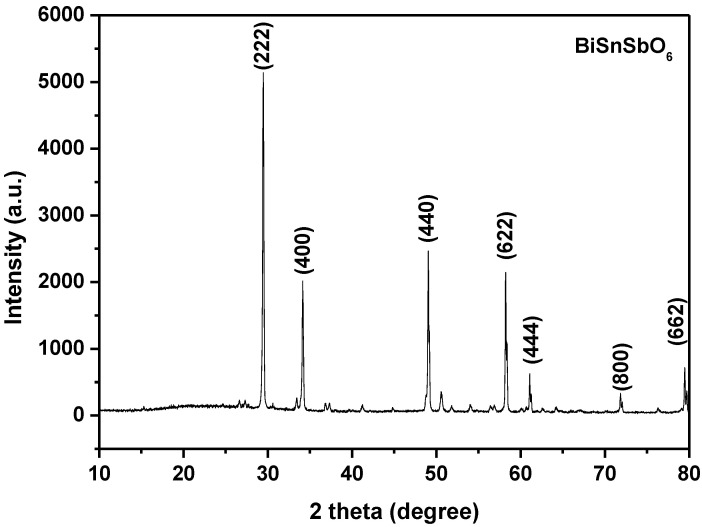
The XRD spectrum of BiSnSbO_6_.

Figure 3 shows the XRD pattern of BiSnSbO_6_ and marks the individual diffraction peaks. The structure of BiSnSbO_6_ was tested by the XRD technique. From the analysis of the results, we might conclude that BiSnSbO_6_ was a single phase and the building block parameters could be equivalently a = b = c = 10.234594 Å. By analyzing the above results, it is shown that BiSnSbO_6_ possesses a pyrochlore structure and the cubic system; simultaneously, the space group was *Fd3m* and the crystallization of BiSnSbO_6_ was good.

**Figure 4:**
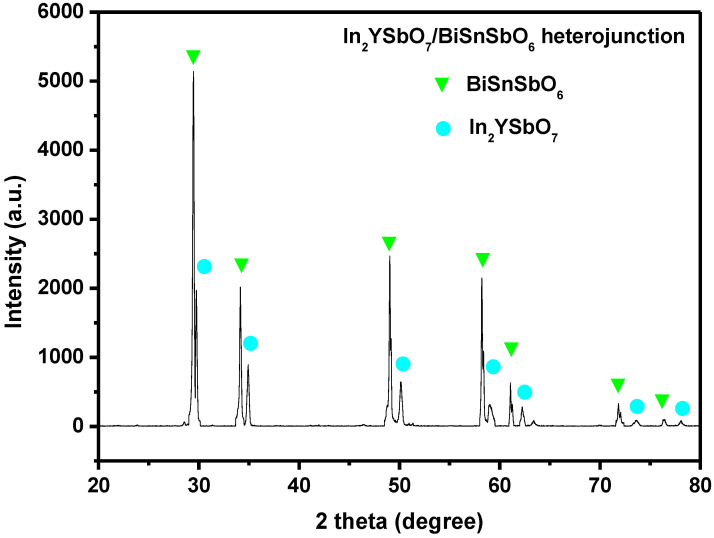
The XRD spectrum of In_2_YSbO_7_/BiSnSbO_6_ heterojunction.

Figure 4 shows the XRD spectrum of IBHP. As can be seen from Figure 4, there were pure single-crystal In_2_YSbO_7_ photocatalyst and pure single-crystal BiSnSbO_6_ photocatalyst. The diffraction peaks of In_2_YSbO_7_ and BiSnSbO_6_ were marked successfully, and no other impurities were found.

**Figure 5:**
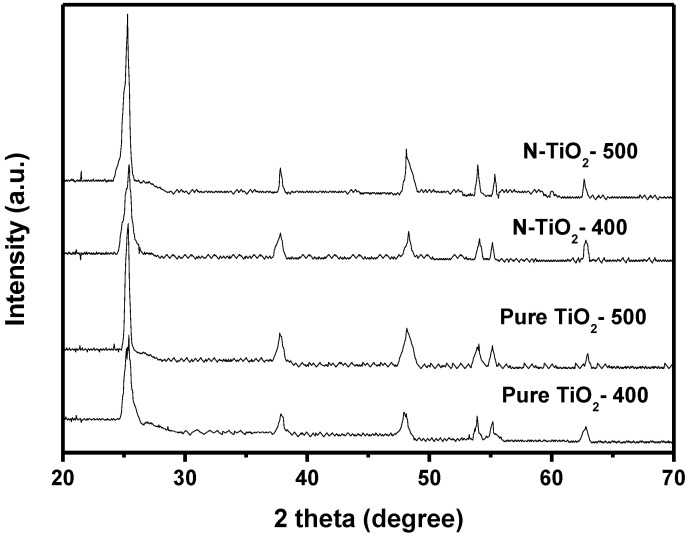
The XRD spectrum of N-TiO_2_ and pure TiO_2_.

Figure 5 shows the X-ray diffraction patterns of N-TiO_2_ and pure TiO_2_. The structure of N-TiO_2_ and pure TiO_2_ was tested by XRD technology. N-TiO_2_-500 was calcined at 500 °C and N-TiO_2_-400 was calcined at 400 °C. It can be seen from Figure 5 that N-TiO_2_ or pure TiO_2_ was mainly composed of anatase phase. 

### 2.2. UV–Vis Diffuse Reflectance Spectra

**Figure 6:**
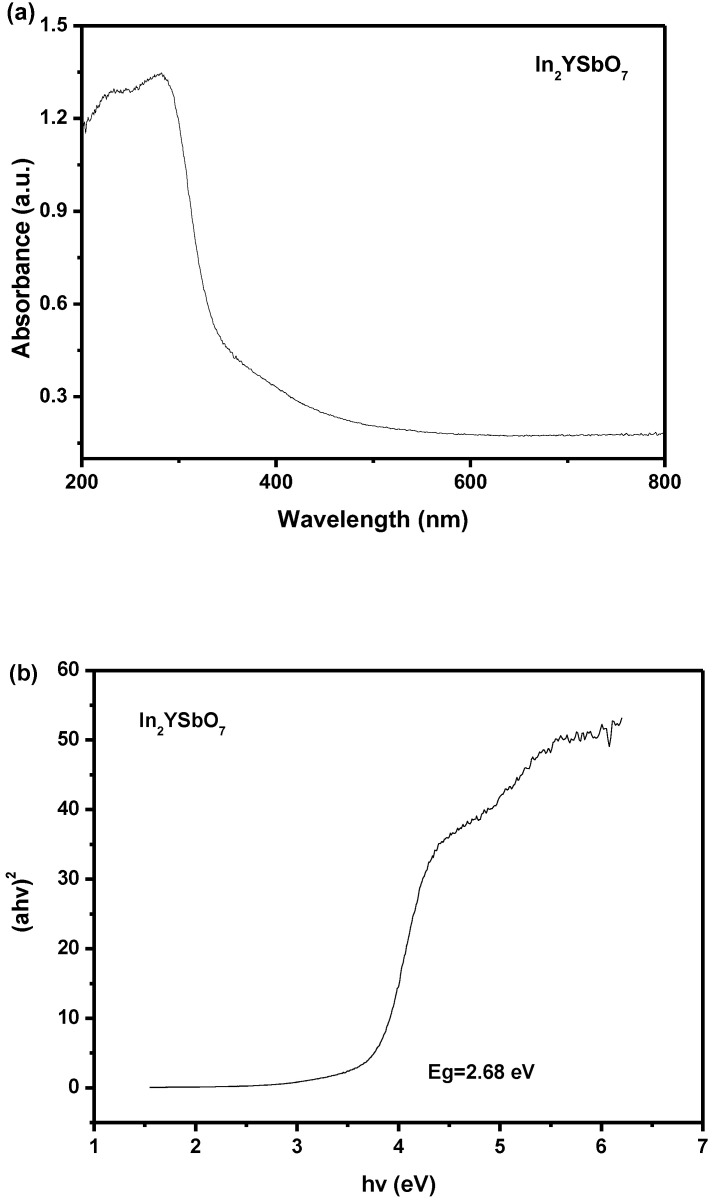
(**a**) UV–vis diffuse reflectance spectra of In_2_YSbO_7_; (**b**) Plot of (*αhν*)^2^ versus *hν* for In_2_YSbO_7_.

The UV–vis diffuse reflectance spectra (U–V DRS) of the In_2_YSbO_7_ sample are listed in Figure 6a,b. The absorption edge of the novel photocatalyst In_2_YSbO_7_ was located in the visible-light region of 503 nm in the spectrum. The band gap energy (BGE) of a semiconductor could be indicated by the intersection between the *hv* axis representing the photon energy and a conjectural line which was described in accordance with the linear part of the absorption edge of the Kubelka–Munk function (1) [59,60].

(1) $$\frac{{\left[1-R_{d}\left(h\nu \right)\right]}^{2}}{2R_{d}\left(h\nu \right)}=\frac{\alpha \left(h\nu \right)}{S}$$

where *S* is the scattering factor, *R_d_* is the diffuse reflectance and *α* is the radiation absorption coefficient. 

The light absorption near the band edges of crystalline semiconductors fitted Equation (2) [61,62]:

*αhν* = A (*hν* − *E_g_*)*^n^* (2)

A was the proportionality constant, *α* was the absorption coefficient, *E_g_* was the band gap and *ν* was the optical frequency. In this equation, n determined the properties of transitions in semiconductors. *E_g_* and *n* could be calculated by the following steps: (1) plot ln (*αhν*) versus ln (*hν − E_g_*) supposed an approximation value of *E_g_*; (2) derive the value of *n* from the slope in this graph; (3) refine the value of *E_g_* by plotting (*αhν*)^1/*n*^ versus *hν* and extrapolating the plot to (*αhν*)^1/*n*^ = 0. According to above methods, the value of *E_g_* for In_2_YSbO_7_ was computed to be 2.68 eV. The reckoning value of *n* was about 0.5 and the optical transition of In_2_YSbO_7_ was a direct transition.

The BGE of In_2_YSbO_7_ was 2.68 eV, the BGE of Bi_3_O_5_I_2_ was 2.02 eV [63] and the BGE of Co-doped ZnO was 2.39 eV [64]. The BGE of every catalyst derived from the above three catalysts was less than 2.69 eV, indicating that above three photocatalysts had strong visible-light catalytic activity.

**Figure 7:**
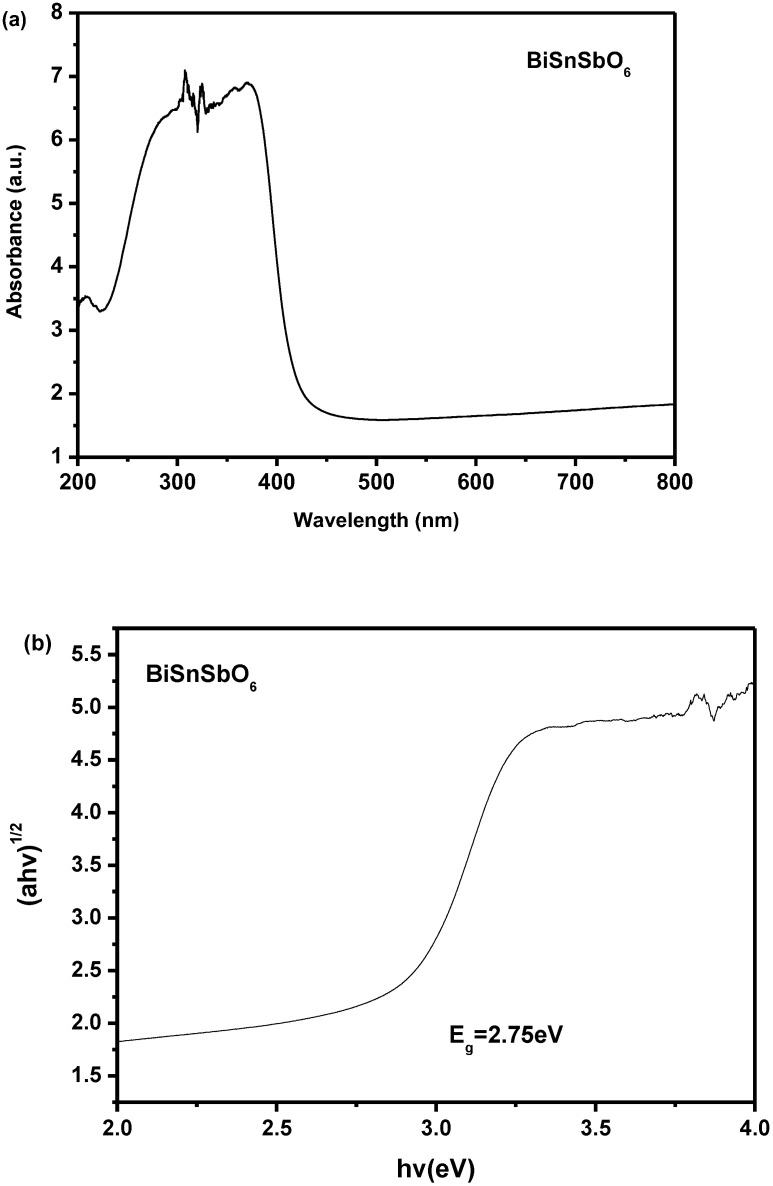
(**a**) The diffused reflection spectrum of BiSnSbO_6_; (**b**) Correlative diagram of (*αhν*)^1/2^ and *hν* for BiSnSbO_6_.

Figure 7a,b shows the U–V DRS of BiSnSbO_6_. According to the results analysis which was based on Figure 7a,b, the Eg value of BiSnSbO_6_ is estimated to be 2.75 eV. The reckoning value of n was about 2, and the optical transition of BiSnSbO_6_ was an indirect transition.

**Figure 8:**
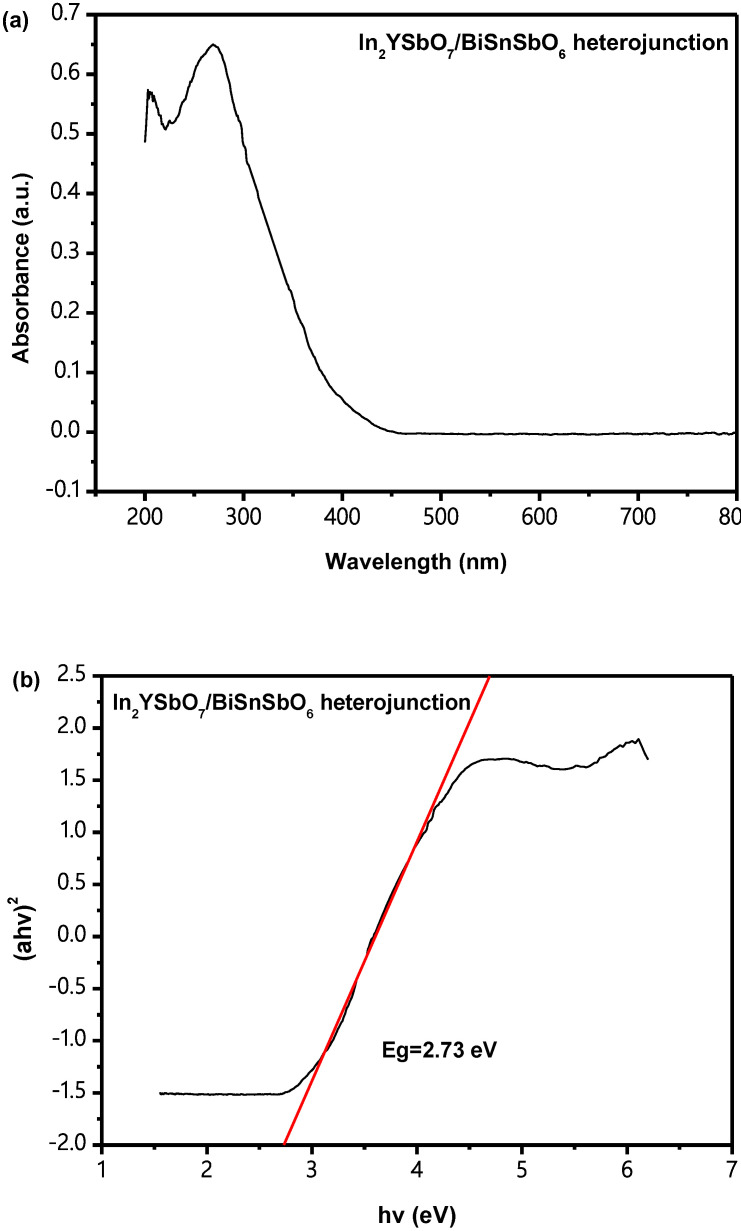
(**a**) The diffused reflection spectrum of In_2_YSbO_7_/BiSnSbO_6_ heterojunction; (**b**) Correlative diagram of (*αhν*)^2^ and *hν* for In_2_YSbO_7_/BiSnSbO_6_ heterojunction.

Figure 8a,b shows the U–V DRS of IBHP. According to the above methods, the value of *Eg* for IBHP was estimated to be 2.73 eV. The reckoning value of n was about 0.5; as a result, the optical transition of IBHP was a direct transition. 

**Figure 9:**
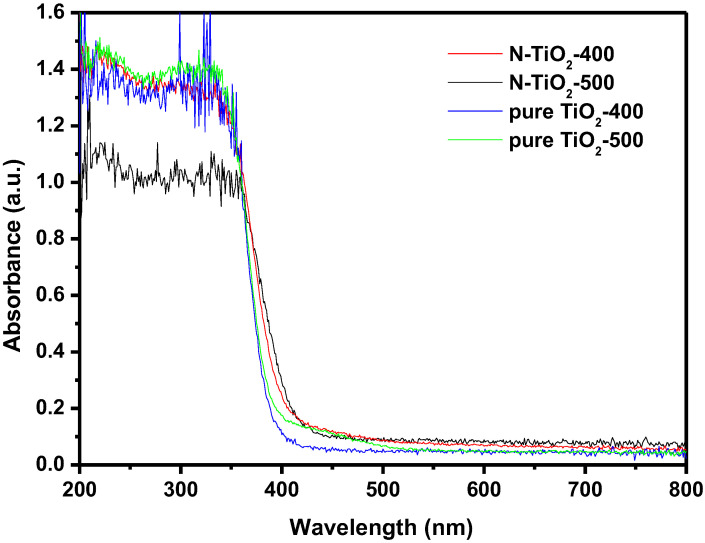
UV–vis absorption spectra of pure TiO_2_ and N-TiO_2_ with different calcination temperatures.

The U–V DRS of TiO_2_ and N-TiO_2_ under different calcination temperatures are shown in Figure 9. In accordance with above procedures and Figure 9, the numerical value of *Eg* for pure TiO_2_ or N-TiO_2_ was calculated to be 3.13 eV or 2.95 eV.

### 2.3. Property Characterization of In_2_YSbO_7_/BiSnSbO_6_ Heterojunction Photocatalyst

**Figure 10:**
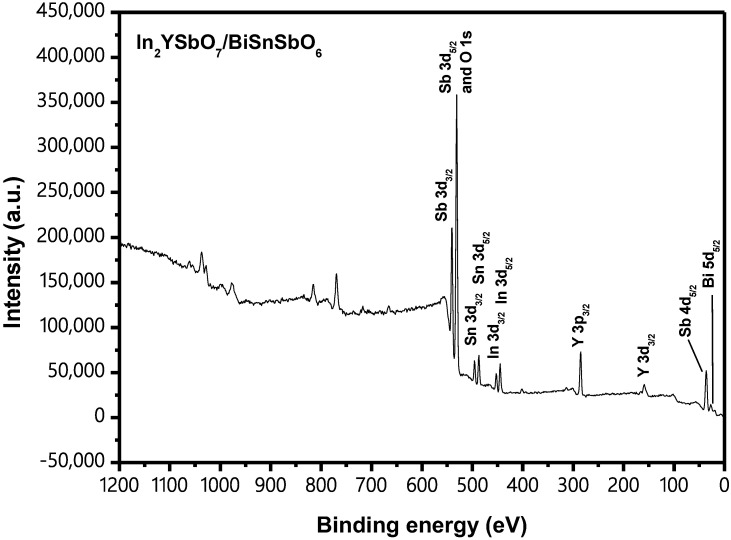
XPS survey spectrum of the IBHP.

**Figure 11:**
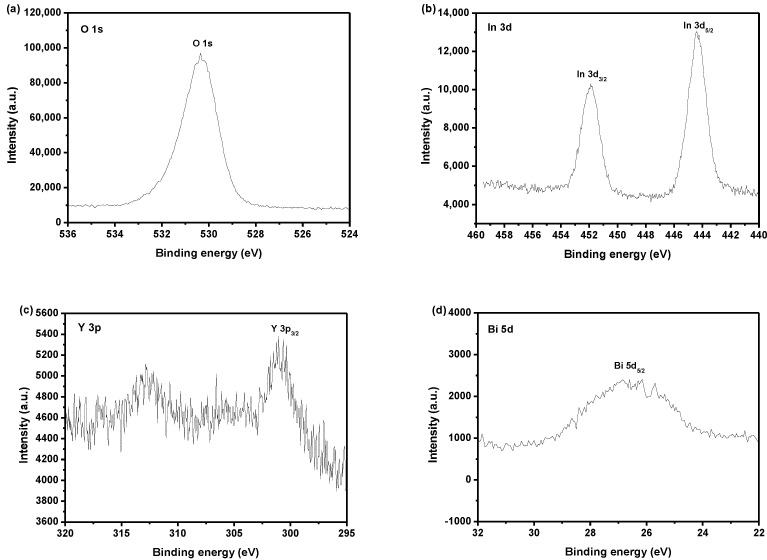

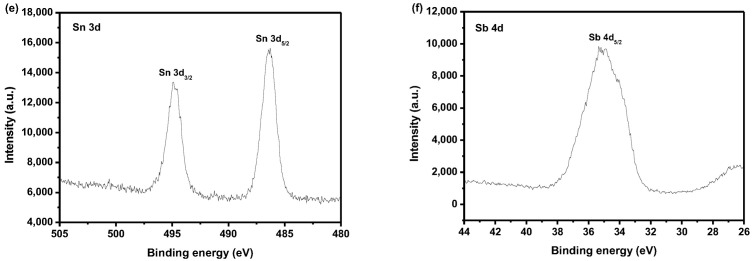
(**a**) XPS spectra of O^2−^ derived from the IBHP; (**b**) XPS spectra of In^3+^ derived from the IBHP; (**c**) XPS spectra of Y^3+^ derived from the IBHP; (**d**) XPS spectra of Bi^3+^ derived from the IBHP; (**e**) XPS spectra of Sn^4+^ derived from the IBHP; (**f**) XPS spectra of Sb^5+^ derived from the IBHP.

Figure 10 shows the X-ray photoelectron spectroscopy (XPS) survey spectrum of IBHP. Figure 11 shows the XPS spectra of O^2−^, In^3+^, Y^3+^, Bi^3+^, Sn^4+^ and Sb^5+^, which derive from IBHP. According to the XPS survey spectrum, the synthetical IBHP comprised the elements of In, Y, Sb, Bi, Sn and O. On the basis of XPS research results, which were shown in Figure 10 and Figure 11, the oxidation state of In, Y, Sb, Bi, Sn and O ions was +3, +3, +5, +3, +4 and −2, respectively. On the basis of research results, the chemical formula of the new sample could be concluded as In_2_YSbO_7_/BiSnSbO_6_. In Figure 11, the O1s peak of O was situated at 530.35 eV. In3d_3/2_ and In3d_5/2_ peaks of In were situated at 451.9 eV and 444.4 eV, respectively. The Y3p_3/2_ peak of Y was situated at 301.05 eV. The position of the Bi5d_5/2_ peak of Bi was situated at 26.85 eV. Sn3d_3/2_ and Sn3d_5/2_ peaks of Sn were situated at 494.95 eV and 486.45 eV. The Sb4d_5/2_ peak of Sb was 35.35 eV. The results of surface elemental analysis showed that the average atomic ratio In:Y:Sb:Bi:Sn:O was 382:193:379:179:186:3681. The atomic ratio of In:Y and Bi:Sn in the sample of IBHP was 1.98:1 and 0.96:1, respectively. The reason that the oxygen value was higher might be due to the large amount of oxygen adsorption on the surface of IBHP. Obviously, the XPS peaks of IBHP did not have shoulder and broadening, which meant that there were no other phases within IBHP. 

**Figure 12:**
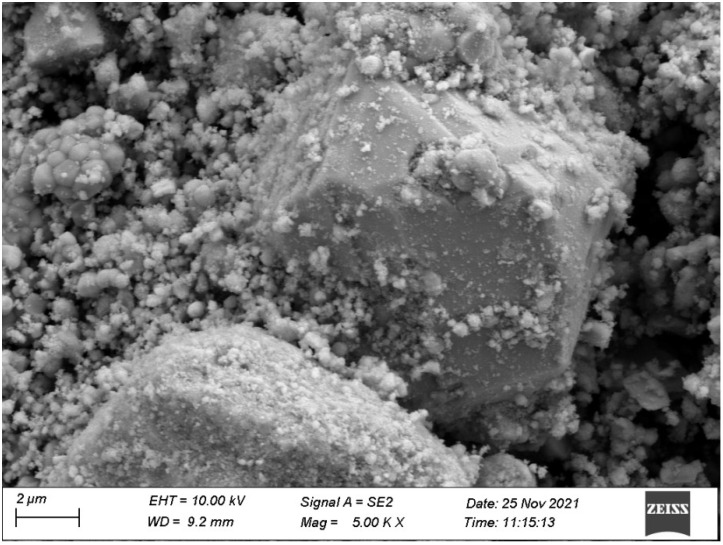
SEM photograph of IBHP.

**Figure 13:**
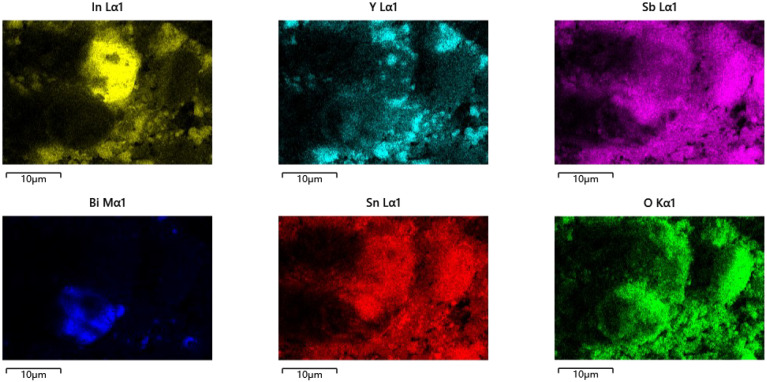
EDS elemental mapping of IBHP (In, Y, Sb, O from In_2_YSbO_7_ and Bi, Sn, Sb, O from BiSnSbO_6_).

As can be seen from Figure 12 and Figure 13, the larger particles belonged to BiSnSbO_6_ and the smaller particles belonged to In_2_YSbO_7_. It is shown in Figure 12 and Figure 13 that the particles of BiSnSbO_6_ were encircled by small particles of In_2_YSbO_7_; two kinds of particles were tightly combined, indicating the successful synthesis of IBHP. 

**Figure 14:**
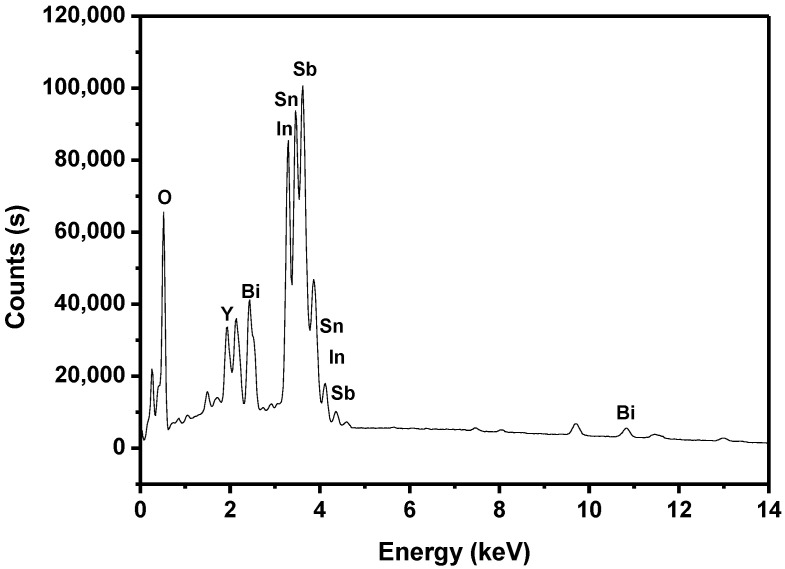
EDS spectrum of IBHP.

The SEM–EDS results shown in Figure 12, Figure 13 and Figure 14 express that there were no other doped elements in the IBHP compound. Meanwhile, the pure phase of In_2_YSbO_7_ was unanimous with the XRD analysis results, as shown in Figure 1. On the basis of Figure 14, the atomic ratio of In:Y:Sb:Bi:Sn:O was 802:397:905:504:5.12:6880. Above results were unanimous with XPS results of IBHP, which are expressed in Figure 10 and Figure 11. The atomic ratio of In_2_YSbO_7_:BiSnSbO_6_ was close to 397:512. According to the above results, under our preparation conditions, we could conclude that IBHP possesses high purity.

**Figure 15:**
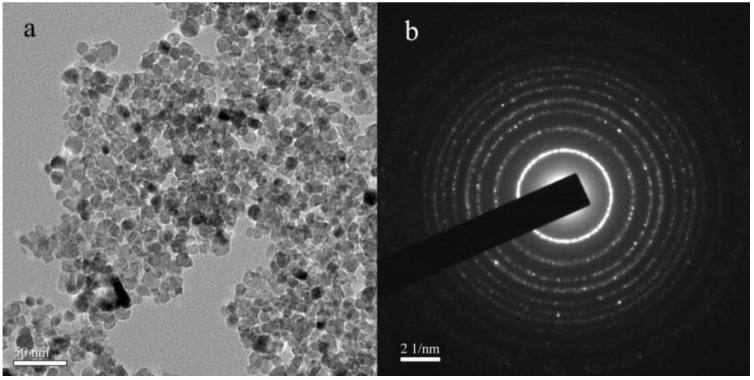
(**a**) The TEM morphology image of N-TiO_2_; (**b**) The selected-area electron diffraction of N-TiO_2_.

Figure 15 displays the TEM morphology image and the selected-area electron diffraction (SAED) of N-TiO_2_. Figure 15 shows that the mean diameter size of the particles of N-TiO_2_ was 10 nm. The limitation of the SAED region was 50 nm; thus, the SAED image of N-TiO_2_ particles is shown as a concentric circle.

### 2.4. Photocatalytic Activity

**Figure 16:**
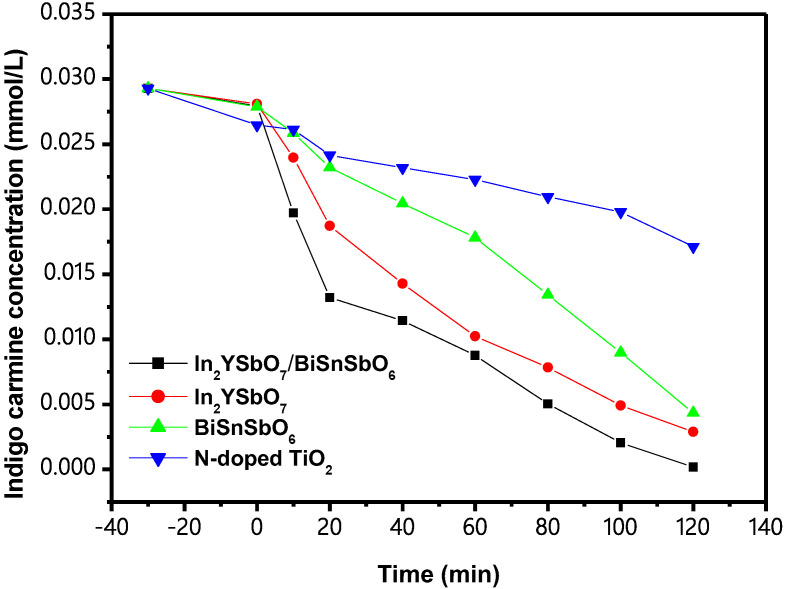
Concentration variation curves of IC during PCD of IC with IBH as a catalyst or with In_2_YSbO_7_ as a catalyst or with BiSnSbO_6_ as a catalyst or with N-TiO_2_ as a catalyst under VLGI.

Figure 16 shows the concentration change curve (CCC) of IC during photocatalytic degradation (PCD) with IBHP or In_2_YSbO_7_, BiSnSbO_6_ or N-TiO_2_ as a catalyst, respectively, under VLGI. In the process of degradation, the concentration of IC in DW gradually decreased with increasing VLGI time. Analysis of the results in Figure 16 showed that the removal rate (ROR) of IC in DW reached 99.42% with a reaction rate of 4.046 × 10^−9^ mol·L^−1^·s^−1^ and the photonic efficiency (PEY) was 0.085% with IBHP after visible-light irradiation of 120 min (VLGI-120M). Other experiments followed the same VLGI time. When we used In_2_YSbO_7_ as a catalyst, the ROR of IC reached 90.14% and the rate of reaction was 3.668 × 10^−9^ mol·L^−1^·s^−1^ and the PEY was 0.077% after VLGI-120M. The ROR of IC within DW reached 85.18% and the rate of reaction was 3.467 × 10^−9^ mol·L^−1^·s^−1^ and the PEY was 0.073% with BiSnSbO_6_ as a catalyst after VLGI-120M. Moreover, the ROR of IC reached 41.57% and the rate of reaction was 1.692 × 10^−9^ mol·L^−1^·s^−1^ and the PEY was 0.036% with N-TiO_2_ as a catalyst after VLGI-120M. In addition, we could summarize from the analysis of the results that the photodegradation efficiency (PDE) of IC in the case of using IBHP was the best; the PDE of IC with In_2_YSbO_7_ as a catalyst was better than that with BiSnSbO_6_ as a catalyst or with N-TiO_2_ as a catalyst. The results show that the photocatalytic activity of IBHP under VLGI was the highest compared with In_2_YSbO_7_, BiSnSbO_6_ and N-TiO_2_. Above results indicate that after VLGI-120M, the ROR of IC, which was degraded with IBHP as a catalyst, was 1.103 times, 1.167 times and 2.392 times higher than that with In_2_YSbO_7_, BiSnSbO_6_ and N-TiO_2_ as a catalyst, respectively. 

**Figure 17:**
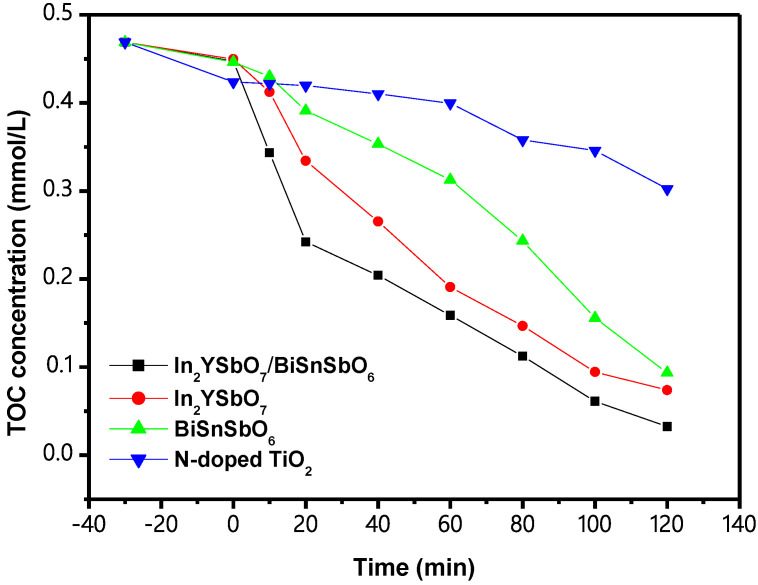
Concentration variation curves of TOC during PCD of IC in DW with IBH as a catalyst or with In_2_YSbO_7_ as a catalyst or with BiSnSbO_6_ as a catalyst or with N-TiO_2_ as a catalyst under VLGI.

Figure 17 shows the CCC of total organic carbon (TOC) at the time of PCD of IC in DW with IBH as a catalyst or with In_2_YSbO_7_ as a catalyst or with BiSnSbO_6_ as a catalyst or with N-TiO_2_ as a catalyst under VLGI. Figure 17 shows that the ROR of TOC within DW reached 93.10%, 84.26%, 80.02% and 35.50%, respectively, after VLGI-120M when IBHP, In_2_YSbO_7_, BiSnSbO_6_ and N-TiO_2_ were used for degrading IC. Finally, by analyzing the results regarding the ROR of TOC at the time of IC degradation, the ROR of TOC with IBHP was the best among the above four catalysts. The obtained results also showed that the ROR of TOC during IC degradation using In_2_YSbO_7_ was much higher than that using BiSnSbO_6_ or N-TiO_2__,_ implying that IBHP possessed the highest mineralization rate during IC degradation compared with In_2_YSbO_7_ or BiSnSbO_6_ or N-TiO_2_.

**Figure 18:**
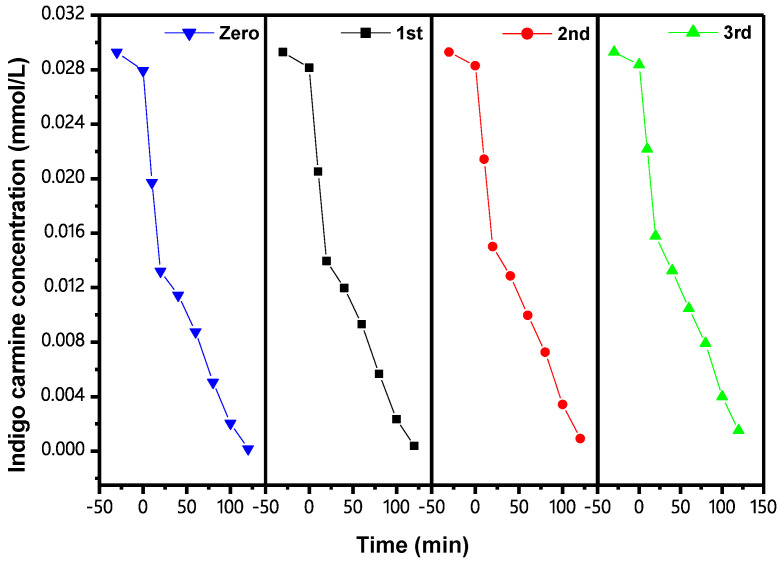
CCC of IC during PCD of IC in DW with IBHP under VLGI for TCDT.

**Figure 19:**
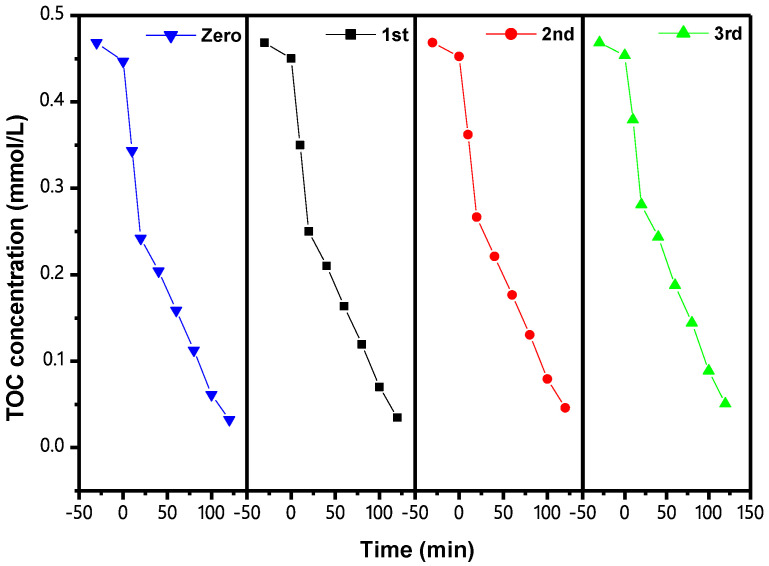
CCC of TOC during PCD of IC in DW with IBHP under VLGI for TCDT.

Figure 18 shows the CCC of IC during PCD with IBHP under VLGI for three cycle degradation tests (TCDT). As can be seen from Figure 18, the ROR of IC reached 98.74%, 96.89% and 94.88%, respectively, after VLGI-120M with IBH as a catalyst. Three cycle experiments were completed for degrading the IC. Figure 19 shows the CCC of TOC during PCD of IC with IBHP under VLGI for TCDT. The experimental data in Figure 19 show that the ROR of TOC was 92.64%, 90.26% and 89.19%, respectively, after VLGI-120M with IBHP when three cycle experiments were completed for degrading IC. The test results shown in Figure 18 and Figure 19 show that IBHP possesses strong stability. 

**Figure 20:**
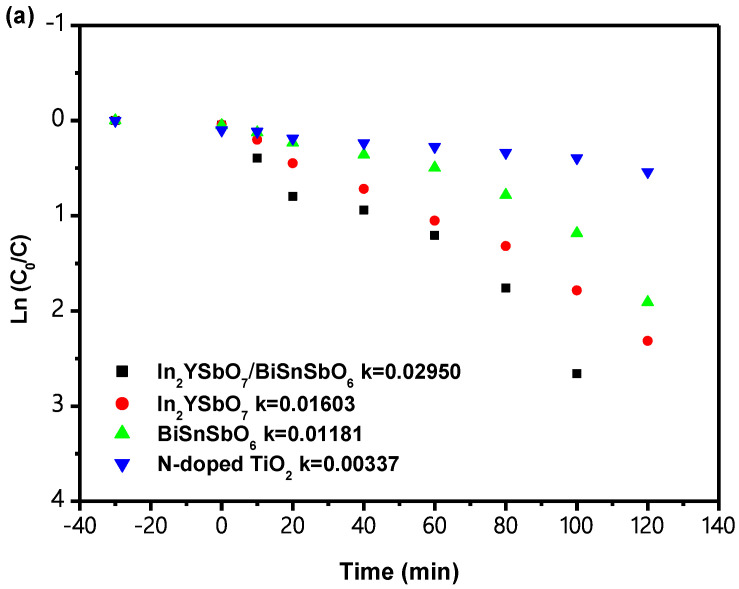

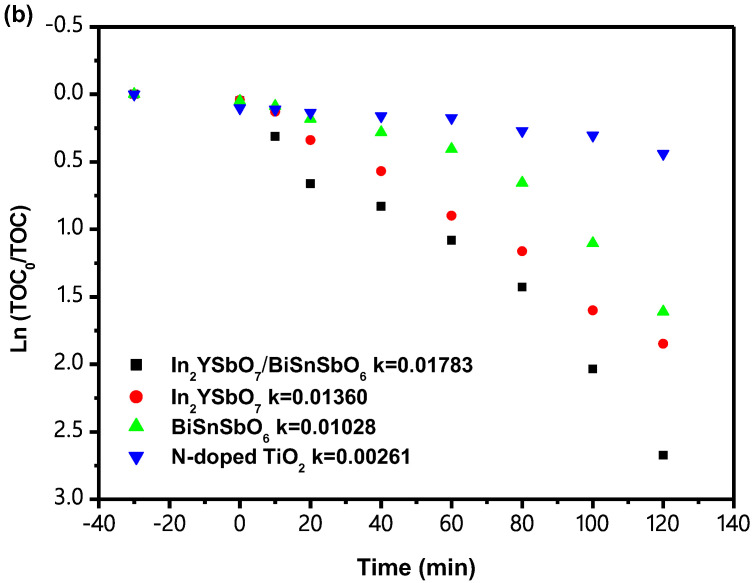
(**a**) Observed FOK plots for the PCD of IC with IBH or In_2_YSbO_7_ or BiSnSbO_6_ or N-TiO_2_ as photocatalyst under VLGI. (**b**) Observed FOK plots for TOC during PCD of IC in DW with IBH or In_2_YSbO_7_ or BiSnSbO_6_ or N-TiO_2_ as photocatalyst under VLGI.

Figure 20 shows the analysis of first-order kinetic (FOK) plots for the PCD of IC with IBH as catalyst or with In_2_YSbO_7_ as a catalyst or with BiSnSbO_6_ as a catalyst or with N-TiO_2_ as a catalyst under VLGI. It can be seen from Figure 20 that the kinetic constant k (KCK), which was from the DCT IC concentration and VLGI time, with IBH, In_2_YSbO_7_, BiSnSbO_6_ or N-TiO_2_ as the catalyst, reached 0.0295 min^−1^, 0.01603 min^−1^, 0.01181 min^−1^ and 0.00337 min^−1^, respectively. The KCK which came from the DCT TOC concentration and VLGI time reached 0.0178 min^−1^, 0.0136 min^−1^, 0.0103 min^−1^ and 0.0026 min^−1^ with IBH, In_2_YSbO_7_, BiSnSbO_6_ or N-TiO_2_ as the catalyst. The *K_TOC_* value obtained during IC degradation was lower than the *K_C_* value obtained when the same catalyst was utilized to degrade IC. At the same time, compared with In_2_YSbO_7_ or BiSnSbO_6_ or N-TiO_2_, IBHP possessed the highest degradation mineralization efficiency for degrading IC.

**Figure 21:**
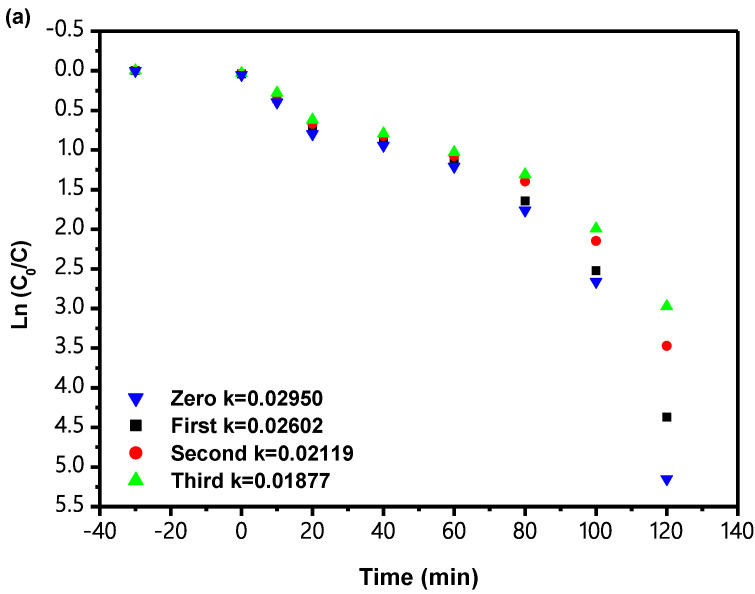

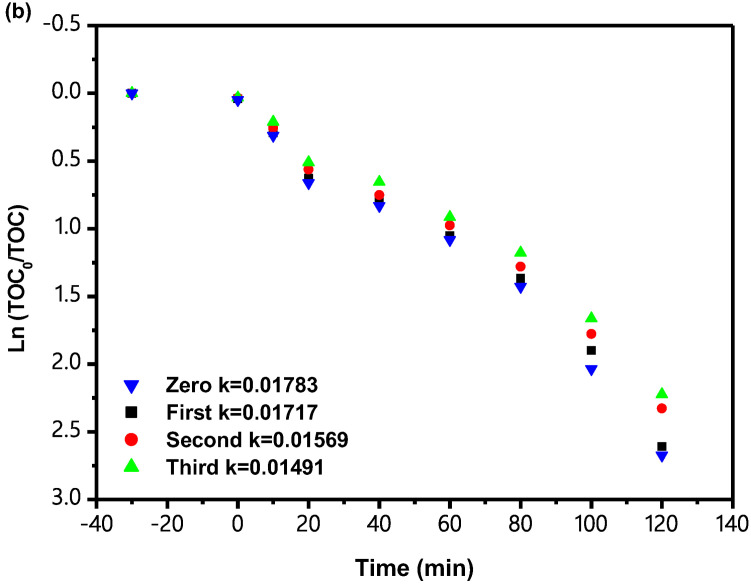
(**a**) Observed FOK plots for the PCD of IC with IBH as photocatalyst under VLGI for three cycle degradation tests. (**b**) Observed FOK plots for TOC during PCD of IC with IBH as photocatalyst under VLGI for three cycle degradation tests.

Figure 21 shows the FOK for the PCD of IC when IBH is used as a catalyst for TCDT under VLGI conditions. According to the results shown in Figure 21, the KCK derived from the DCT IC concentration and VLGI time with IBHP for TCDT achieved 0.02602 min^−1^, 0.02119 min^−1^ and 0.01877 min^−1^. In addition, the KCK which came from the DCT TOC concentration and VLGI time with IBH as a photocatalyst for TCDT reached 0.01717 min^−1^, 0.01569 min^−1^ and 0.01491 min^−1^. The results of the analysis are shown in Figure 20 and Figure 21; the PCD of IC in DW with IBH as a photocatalyst under VLGI conformed to first-order reaction kinetics. 

The analysis results in Figure 21 show that after TCDT, under VLGI, with IBHP, the ROR of IC was reduced by 3.86%, and the ROR of TOC was reduced by 3.45%. Above results indicate that there was no significant difference in the degradation efficiency of TCDT, and the catalyst structure of IBHP was stable. 

**Figure 22:**
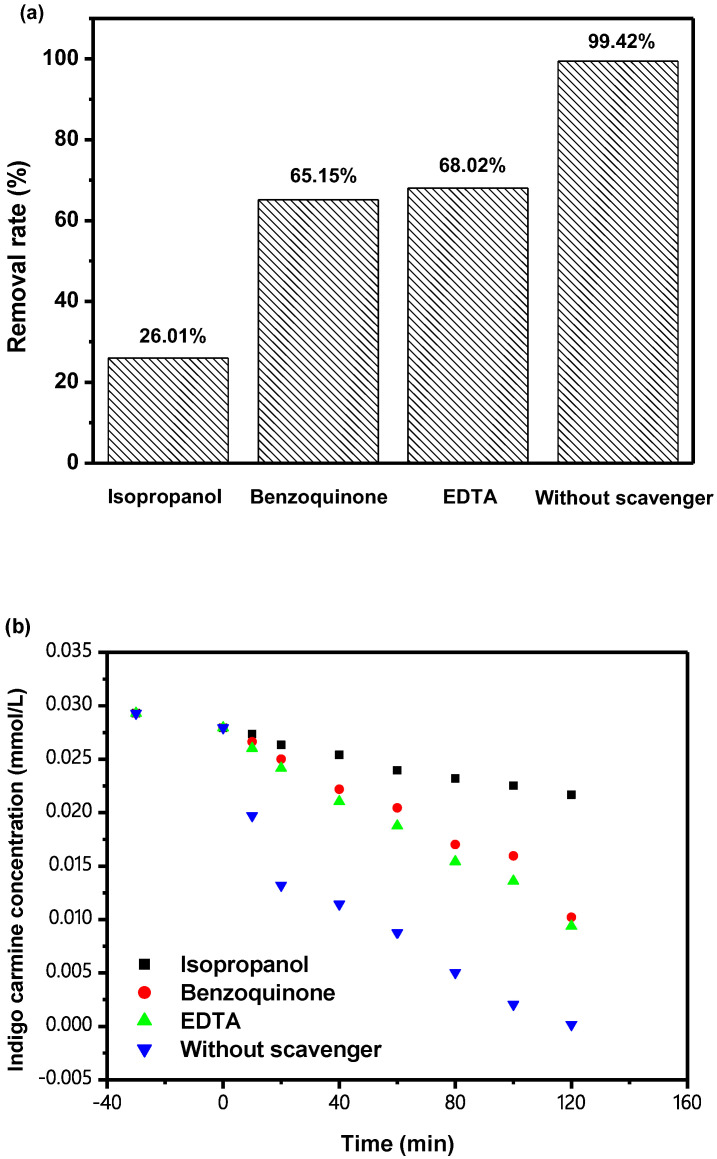
(**a**) Effect of different RS on ROR of IC with IBH as catalyst under VLGI; (**b**) Effect of different RS such as BQ, IPA or EDTA on the removal efficiency of IC with IBHP under VLGI.

Figure 22 shows the effect of adding different radical scavengers (RS) such as benzoquinone (BQ) or isopropanol (IPA) or ethylenediamine tetra-acetic acid (EDTA), respectively, on the ROR of IC with IBHP under VLGI. The experiments first mixed different RS in IC solution to determine the active species during IC degradation. IPA was utilized for capturing hydroxyl radicals (^•^OH), and BQ was utilized for capturing superoxide anions (^•^O_2_^−^), and ethylenediamine tetra-acetic acid (EDTA) was utilized for capturing holes (h^+^). The planned concentration of IPA or EDTA or BQ was 0.15 mmol L^−1^, and the addition amount of EDTA or IPA or BQ was 1 mL. As shown in Figure 22, when the BQ, IPA or EDTA was mixed in the IC solution, the ROR of IC decreased by 73.41%, 34.27% and 31.4%, respectively, compared with the ROR of IC derived from the control group. Therefore, we can draw the conclusion that ^•^OH, ^•^O_2_^−^ and h^+^ were all active radicals during IC degradation. As shown in Figure 22, ^•^OH in IC solution showed a key role in the degradation of IC with IBHP under VLGI. Through experiments, compared with superoxide anions or holes, hydroxyl radicals possessed the largest oxidative removal capacity for IC in DW. The oxidizing ability of the three types of oxidative radical for degrading IC was, from high to low: hydroxyl radicals > superoxide anions > holes.

**Figure 23:**
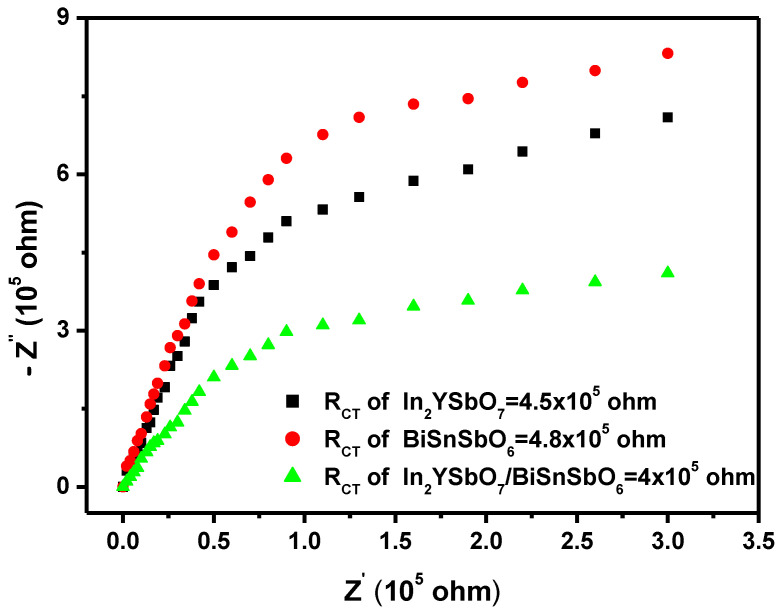
Nyquist impedance plots of IBHP or In_2_YSbO_7_ photocatalyst or BiSnSbO_6_ photocatalyst.

Figure 23 shows the Nyquist impedance plots of IBHP or In_2_YSbO_7_ photocatalyst or BiSnSbO_6_ photocatalyst. The Nyquist impedance map shows the PE transport processes and PH transport processes between the solid and the electrolyte for the synthetical photocatalyst. The smaller the radius of the arc is, the higher the transmission efficiency of the photocatalyst is. Figure 23 indicates that the arc radius is in the order: BiSnSbO_6_ > In_2_YSbO_7_ > IBHP. Above results show that the preparative IBHP possesses high separation efficiency of PE and PH and fast interface charge transfer capability. The charge transfer resistance (R_CT_, which was calculated according to the diameter of a semicircle) of BiSnSbO_6_, In_2_YSbO_7_ and IBHP was 4.8 × 10^5^ ohm, 4.5 × 10^5^ ohm or 4.0 × 10^5^ ohm, based on the Nyquist plot displayed in Figure 23.

### 2.5. Degradation Mechanism Analysis

**Figure 24:**
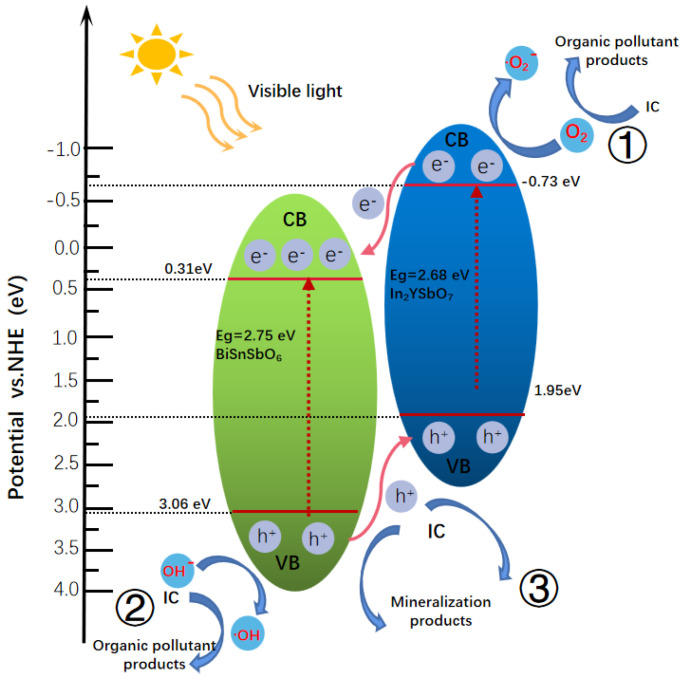
Possible PCD mechanism of IC with IBHP under VLGI.

Figure 24 displays the presumed photocatalytic degradation (PCD) mechanism of IC with IBH as a catalyst under VLGI. The potentials of the conductor band (CB) and valence band (VB) of the semiconductor can be computed according to the following Formulas (3) and (4) [65]:

*E_CB_* = *X* − *E^e^* − 0.5*E_g_* (3)

*E_VB_* = *E_CB_* + *E_g_* (4)

In the formulas, *X* is the electronegativity of the semiconductor, *E^e^* is the energy of free electrons on the hydrogen scale and *E_g_* is the band gap. Based on above formulas, it can be seen that the VB potential and CB potential of In_2_YSbO_7_ are about 1.95 eV and −0.73 eV, respectively. For BiSnSbO_6_, the VB potential and CB potential are about 3.06 eV and 0.31 eV, separately. It was observed that both In_2_YSbO_7_ and BiSnSbO_6_ could absorb visible light; simultaneously, PE and PH could be generated when the IBHP was under the condition of VLGI. Due to the fact that the redox potential position of CB for In_2_YSbO_7_ (−0.73 eV) was more negative than that of BiSnSbO_6_ (0.31 eV), the PE on the CB of In_2_YSbO_7_ was diverted to the CB of BiSnSbO_6_. Moreover, the redox potential position of VB for BiSnSbO_6_ (3.06 eV) was more positive than that of In_2_YSbO_7_ (1.95 eV), so the PH on the VB of BiSnSbO_6_ was transferred to the VB of In_2_YSbO_7_. Therefore, IBHP produced by the coupling of In_2_YSbO_7_ and BiSnSbO_6_ can efficiently decrease the recombination rate of PE and PH, thereby reducing the internal resistance, extending the lifetime of the PE and the PH; as a result, the interfacial charge metastasis efficiency is improved [66]. In addition, the CB potential of In_2_YSbO_7_ was −0.73 eV, which was more nonpositive than that of O_2_/^•^O_2_^−^ (−0.33 V), demonstrating that the electrons in the CB of In_2_YSbO_7_ could assimilate subaqueous soluble oxygen for producing ^•^O_2_^−^, which coan degrade IC efficiently, as shown in Path 1. Meanwhile, the VB potential of BiSnSbO_6_ was 3.06 eV, which was more nonnegative than OH^−^/^•^OH (2.38 V), illustrating that the PH in the VB of BiSnSbO_6_ could oxidize H_2_O or OH^−^ into ^•^OH, which can degrade IC effectively; this is displayed in Path 2. The PH in the VB of In_2_YSbO_7_ or BiSnSbO_6_ could immediately oxidize IC for degradation of IC due to their powerful oxidizing capability, as shown in Path 3. To sum up, the excellent photocatalytic activity of IBHP toward IC degradation is mainly attributed to the higher separation efficiency of the PE and the PH, which was induced by IBHP.

**Figure 25:**
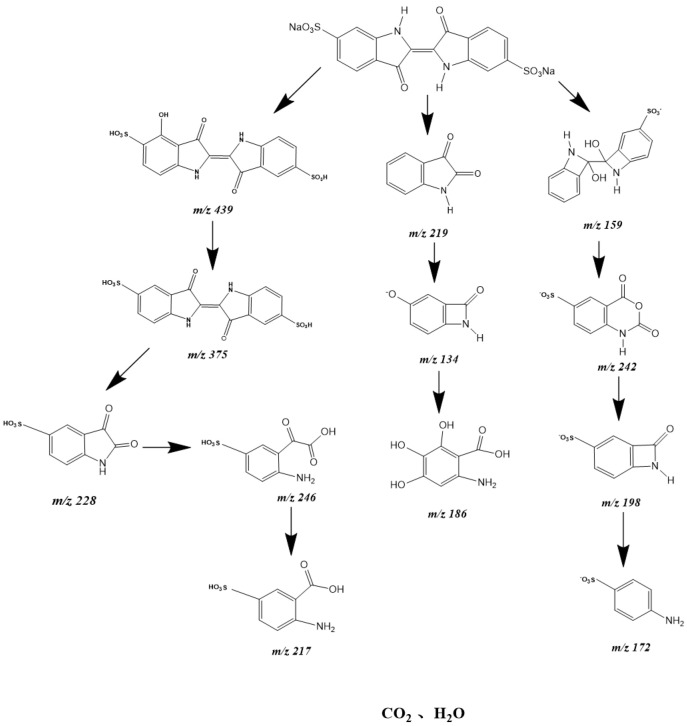
Possible PCD pathway scheme for IC under VLGI with IBHP.

In order to research the degradation mechanism of IC, LC–MS was used to analyze the intermediate products during the IC degradation process. The intermediate products were isatin (*m*/*z* = 219), 5′,6′-dihydroxy-3,3′-dioxo-[2,2′-biindolinylidene]-5-sulfonic acid (*m*/*z* = 375), diethyl oxalate (m/*z* = 146), ethyl oxamate (*m*/*z* = 117), 4-hydroxy-3,3′-dioxo-[2,2′-biindolinylidene]-5,5′-disulfonic acid (*m*/*z* = 439), C_8_H_4_NO_6_S (*m*/*z* = 242), 2,3-dioxoindoline-5-sulfonic acid (m/*z* = 228), 2-(2-amino-5-sulfophenyl)-2-oxoacetic acid (*m*/*z* = 246), 2-amino-5-sulfobenzoic acid (*m*/*z* = 217), 6-amino-2,3,4-trihydroxybenzoic acid (*m*/*z* = 186), oxalic acid, aniline and acetic acid. The analysis of detected intermediate products showed possible PCD paths of the IC. Figure 25 shows a possible PCD pathway scheme with IBHP for IC degradation under the condition of VLGI. As shown in Figure 25, the hydroxylation reaction, oxidation reaction, methylation reaction, decarboxylation reaction and desulfonation reaction were achieved. 

## 3. Experimental Section

### 3.1. Materials and Reagents

P-benzoquinone (BQ, C_6_H_4_O_2_, purity ≥ 98.0%) was chemical grade (Sinopharm Group Chemical Reagent Co., Ltd., Shanghai, China). Ethylenediaminetetraacetic acid (EDTA, C_10_H_16_N_2_O_8_, purity = 99.5%) and isopropyl alcohol (IPA, C_3_H_8_O, purity ≥ 99.7%) were analytical grade. Absolute ethanol (C_2_H_5_OH, purity ≥ 99.5%) was compliant with American Chemical Society Specifications (Aladdin Group Chemical Reagent Co., Ltd., Shanghai, China). IC (C_16_H_8_N_2_Na_2_O_8_S_2_, purity ≥ 98%) was gas chromatography grade (Tianjin Bodi Chemical Co., Ltd., Tianjin, China). Ultra-pure water (18.25 MU cm) was used throughout the work.

### 3.2. Synthesis of N-Doped TiO_2_

Using tetrabutyl titanate as a precursor and ethanol as a solvent, nitrogen-doped titanium dioxide catalyst was synthesized and the sol-gel method was used for preparation. The work sequence was as following: Firstly, 17 mL tetrabutyl titanate and 40 mL anhydrous ethanol were combined as solution A; 40 mL of absolute ethanol, 10 mL of glacial acetic acid and 5 mL of double-distilled water were mixed as solution B; subsequently, ammonia water with a N/Ti ratio of 8 mol% was put into the clear colloidal suspension, which was formed by adding solution A dropwise to solution B under strong magnetic stirring and continuous magnetic stirring for 1 h. Xerogel was produced after 2 days of aging. The xerogel was ground into a powder and calcined at 500 °C for 2 h; finally, the mixed powder was sieved with a vibrating sieve after pulverization to obtain N-TiO_2_ powder. 

### 3.3. Synthesis of In_2_YSbO_7_/BiSnSbO_6_ Heterojunction Photocatalyst

First, 0.30 mol/L In (NO_3_)_3_·5H_2_O, 0.15 mol/L Y(NO_3_)_3_·6H_2_O and 0.15 mol/L SbCl_5_ were blended and continuously stirred for 20 h. The solution was then put into an autoclave and heated at 200 °C for 15 h. Hereafter, under the condition of N_2_ ambience, the mixed compounds were calcined at 800 °C for 10 h in a tube furnace with a ramp rate of 8 °C/min. In_2_YSbO_7_ powder was then obtained. After the above operation, 0.15 mol/L Bi(NO_3_)_3_·5H_2_O, 0.15 mol/L SnCl_4_·5H_2_O and 0.15 mol/L SbCl_5_ were blended and continuously stirred for 20 h and heated at 200 °C for 15 h. Then, the resulting powder was calcined at 780 °C for 10 h at 8 °C/min under an ambience of N_2_. Thus, BiSnSbO_6_ powder was obtained.

IBHP was made by mixing 800 mg of In_2_YSbO_7_ with 30 wt% (240 mg) of BiSnSbO_6_ in 200 mL of octanol (C_8_H_18_O) and then the above mixed compounds were dispersed in an ultrasonic bath for 1 h with a simple solvothermal method [67]. Subsequently, the admixture was heated and refilled at 140 °C for 2 h under the condition of intense agitation to improve the adhesion of BiSnSbO_6_ to the surface of In_2_YSbO_7_ nanoparticles to form IBHP. After cooling to room temperature, the outcomes were gathered by centrifugation and washed several times with an n-hexane/ethanol mixture. The refined powders were kept arid in a vacuity dryer at 60 °C for 6 h and then deposited into a dry container. Thus, IBHP was obtained.

### 3.4. Characterizations

The prepared pure crystal samples were tested by an X-ray diffractometer (XRD, Shimadzu, XRD-6000, Cu Kα radiation, Kyoto, Japan). The microstructure and morphology of the prepared products were represented by scanning electron microscopy (SEM, FEI, Quanta250, Lincoln, NE, USA), and the component content of the products was detected by energy-dispersive spectroscopy. Diffuse reflectance spectra of the synthetic substance were acquired using a UV–vis spectrophotometer (UV–vis DRS, Shimadzu, UV-3600). The surface chemical composition content and the elemental valence of the synthetic substance were detected by an X-ray photoelectron spectrograph (XPS, UlVAC-PHI, PHI 5000 VersaProbe, Kyoto, Japan). TEM (JEM-200CX, JEOL Corporation, Akishima, Japan) was used for detecting the morphology image and the SAED of N-TiO_2_.

### 3.5. Photoelectrochemical Experiments

For electrochemical impedance spectroscopy (EIS) experiments, the practical instrument was a CHI660D electrochemical station with three standard electrodes (Shanghai Chenhua Instrument Co., Ltd., Shanghai, China). In this system, the working electrode was the prepared catalyst, the counter electrode was a platinum plate and the reference electrode was the commercial Ag/AgCl electrode. The electrolyte was Na_2_SO_4_ aqueous solution (0.5 mol/L), and photochemical measurements were performed using a 500 W Xe lamp with a UV cut-off filter as the visible-light source. The working electrode was prepared as following: after ultrasonic treatment for 1 h, 0.03 g sample and 0.01 g chitosan were dissolved in 0.45 mL dimethylformamide to form a solution. Subsequently, it was dropped onto a 10 mm × 20 mm indium tin oxide conductive glass. Finally, the working electrode was dried at 80 °C for 10 min. The frequency range for the EIS was from 0.01 Hz to 100 kHz.

### 3.6. Experimental Setup and Procedure

The working temperature was kept at 20 °C by using circulating cooling water and a photocatalytic reactor (XPA-7, Xujiang Power Plant, Nanjing, China). Imitation of sunlight was achieved by using a 500 W xenon lamp with a 420 nm cut-off filter. Twelve quartz tubes containing 480 mL of the experimental solution were used. The content of In_2_YSbO_7_ or BiSnSbO_6_ or IBHP was 0.75 g/L; moreover, the concentration of IC was 0.0293 mmol/L. The IC concentration of 1.2 mmol/L was the residual concentration of DW after biodegradation. During the experiment, a UV–vis spectrophotometer (Shimadzu, UV-2450) was used to detect the residual concentration of IC by using 3 mL of the catalyst-filtered suspension solution, which was extracted periodically. Prior to VLGI, the suspension that contained the photocatalyst and IC was stirred magnetically in the dark for 30 min to ensure sufficient adsorption of IC and atmospheric oxygen within the photocatalyst; as a result, the adsorptive saturated suspension was established. Under visible-light illumination, the suspension was stirred at 500 rpm.

Degradation data of IC in the experimental procedure were detected by the TOC analyzer. For testing the concentration of TOC during PCD of IC, potassium acid phthalate (KHC_8_H_4_O_4_) or anhydrous sodium carbonate was used as the standard reagent. Potassium hydrogen phthalate standard solutions with defined carbon concentrations were prepared for calibration. TOC concentration was determined with six samples and every sample contained 45 mL of reaction solution.

Determination of IC and intermediate products were carried out by liquid chromatography–mass spectrometry (LC-MS, Thermo Quest LCQ Duo, Thermo Fisher Scientific Corporation, MA, USA. Beta Basic-C18 HPLC column: 150 × 2.1 mm, ID of 5 μm). A total of 20 µL of the reaction solution was injected into the LC–MS system. The reaction solution contained 60% methanol and 40% ultrapure water at a flow rate of 0.2 mL/min. The mass spectrometry conditions included a capillary temperature of 27 °C, a voltage of 19.00 V, a spray voltage of 5000 V and a constant sheath gas flow. Spectra were acquired over the *m*/*z* range of 50 to 600. 

The incident photon flux after VLGI measured with a radiometer was 4.76 × 10^−6^ Einstein L^−1^ s^−1^. By regulating the distance between the photoreactor and the xenon arc lamp, the incident photon flux on the photoreactor was altered.

The calculation method of photon efficiency was described by the following Formula (5):

*ϕ* = *R/I_o_* (5)

where *ϕ* is the photonic efficiency (%) and *R* is the degradation rate of IC (mol L^−1^ s^−1^) and *I_o_* is the incident photon flux (Einstein L^−1^ s^−1^).

## 4. Conclusions

Firstly, In_2_YSbO_7_ showing intense photocatalytic activity was manufactured by a solvothermal method. IBHP was synthesized by a solvothermal method for the first time. SEM–EDS, XRD, an UV–vis spectrophotometer and XPS were used to investigate the photophysical properties and photocatalytic properties of the prepared photocatalysts. The experimental results displayed that In_2_YSbO_7_ was a pure phase with a pyrochlore structure and a cubic crystal system by the space group *Fd3m*. The lattice parameter and the band gap of In_2_YSbO_7_ were a = 11.102698 Å and 2.68 eV, respectively. IBHP was certified to be an effective catalyst for the removal of IC in the DW. After VLGI-120M, the ROR of IC and TOC reached 99.42% and 93.10%, respectively. The removal rate of IC with IBHP was 1.103 times or 1.167 times or 2.392 times higher than that with In_2_YSbO_7_ as a catalyst or with BiSnSbO_6_ as a catalyst or with N-TiO_2_ as a catalyst after VLGI-120M. Therefore, IBHP was an efficient photocatalyst for treating DW or surface water which was contaminated by IC. In the end, the possible PCD pathways for IC were speculated.

## Figures and Tables

**Table 1:** Structural parameters of In_2_YSbO_7_ synthesized by solvothermal method.

Atom	x	y	z	Occupation Factor
In	0	0	0	1
Y	0.5	0.5	0.5	0.5
Sb	0.5	0.5	0.5	0.5
O(1)	−0.185	0.125	0.125	1
O(2)	0.125	0.125	0.125	1
